# 346. Evaluation of the Safety Profile of Cefazolin Monotherapy in Outpatient Parenteral Antibiotic Antimicrobial Therapy at a Large Academic Medical Center

**DOI:** 10.1093/ofid/ofad500.417

**Published:** 2023-11-27

**Authors:** Nicolas Poux, Eric M Gillett, Catherine Franklin, Mark J Estano, Charles Dewan, Kate Sheedy, Brandon Dionne, David W Kubiak, Jeffrey C Pearson, Brian T Chan

**Affiliations:** Harvard Medical School, Brookline, Massachusetts; Brigham and Women's Hospital, Cambridge , Massachusetts; Brigham and Women's Hospital, Cambridge , Massachusetts; Brigham and Women's Hospital, Cambridge , Massachusetts; Brigham and Womens Hospital, Boston, Massachusetts; Brigham and Women's Hospital, Cambridge , Massachusetts; Brigham and Women's Hospital, Cambridge , Massachusetts; Brigham & Women's Hospital, Boston, Massachusetts; Brigham and Women's Hospital, Cambridge , Massachusetts; Brigham and Women's Hospital, Cambridge , Massachusetts

## Abstract

**Background:**

The Infectious Diseases Society of America recommends serial safety lab monitoring during outpatient parenteral antimicrobial therapy (OPAT) to assess treatment response and prevent adverse events. However, these recommendations do not address the safety profile of specific antimicrobial agents or the ideal frequency of monitoring. Previous studies, using cohorts of fewer than 300 patients, report adverse event rates of less than 5% with outpatient cefazolin. It is possible that serial lab monitoring for patients on cefazolin monotherapy may represent unnecessary work and increase costs with minimal patient benefit.

**Methods:**

A retrospective chart review was performed on patients treated with cefazolin monotherapy in the OPAT program of Brigham and Women’s Hospital (Boston, MA, USA) from February 2018 to February 2023. Data from weekly safety labs were examined during OPAT for the following abnormalities: change in creatinine (absolute increase of 0.5 mg/dL or relative increase ≥50%), ALT > 100 units/L, neutropenia (absolute neutrophil count < 1000 cells/mm^3^), eosinophilia ( >500 cells/mm^3^), and thrombocytopenia (< 100K/uL or relative decrease ≥50%). Other treatment complications adjudicated from chart review included antibiotic treatment changes, readmission, or mortality during OPAT.

**Results:**

Among the 420 patients receiving cefazolin in OPAT, the median age was 62 (IQR: 49-71) years; 168 patients (40%) were women, and 62 (15%) were referred from oncology or transplant services. Twenty-four (6%) patients had at least one lab abnormality. Of these, 13 (3%) required a treatment change or had readmission or death. Eight (2%) cases were related to a lab abnormality, while five were due to other factors either unrelated to antibiotic therapy or apparent on clinical examination (e.g., rash).

**Conclusion:**

In an OPAT program at a large academic medical center, weekly lab monitoring altered clinical decision-making for 2% of patients on cefazolin monotherapy. It may be reasonable to reduce the frequency of or eliminate routine lab monitoring for these patients to reduce the cost of healthcare services. Future studies are indicated to evaluate the clinical impact of less frequent lab monitoring for OPAT patients on cefazolin monotherapy.

**Disclosures:**

**David W. Kubiak, PharmD, BCPS, BCIDP, FIDSA**, Astellas Pharma, Inc.: Advisor/Consultant|AVIR Pharma Inc: Advisor/Consultant|Cidara Therapeutics: Advisor/Consultant|Shionogi Inc: Grant/Research Support

